# Feature Quantification and Abnormal Detection on Cervical Squamous Epithelial Cells

**DOI:** 10.1155/2015/941680

**Published:** 2015-03-22

**Authors:** Mingzhu Zhao, Lei Chen, Linjie Bian, Jianhua Zhang, Chunyan Yao, Jianwei Zhang

**Affiliations:** ^1^College of Computer Science and Technology, Zhejiang University of Technology, Hangzhou 310023, China; ^2^Department of Informatics, University of Hamburg, 22527 Hamburg, Germany

## Abstract

Feature analysis and classification detection of abnormal cells from images for pathological analysis are an important issue for the realization of computer assisted disease diagnosis. This paper studies a method for cervical squamous epithelial cells. Based on cervical cytological classification standard and expert diagnostic experience, expressive descriptors are extracted according to morphology, color, and texture features of cervical scales epithelial cells. Further, quantificational descriptors related to cytopathology are derived as well, including morphological difference degree, cell hyperkeratosis, and deeply stained degree. The relationship between quantified value and pathological feature can be established by these descriptors. Finally, an effective method is proposed for detecting abnormal cells based on feature quantification. Integrated with clinical experience, the method can realize fast abnormal cell detection and preliminary cell classification.

## 1. Introduction

Cervical cancer is one of the most malignant tumors that hazard women's health, and the morbidity of cervical cancer is rising consistently in recent years. Generally, the incubation period before the real formation of cervical cancer is long, and the early detection and confirmation can prevent it from further deteriorating.

Due to the comparatively easy curing of cervical cancer in the early stage, manual detection and identification become necessary. Moreover, fatigue and subjective factors may contribute to the improper diagnosis of cervical cancer [1–3]. Thus, it is necessary to build an efficient and highly accurate automatic diagnosis system.

The methods of computer image processing and analysis are applied to the study of cervical cell images, which mainly concerns the preprocessing of original images, cell feature extractions, classification of data, and the diagnosis outcome. There are many related works in the literature. In [4], a bottom-up searching method is applied to automatically examine cancer cells. It used 40 images, containing 149 cells, to validate the high performance of their proposed method. By using the method, all cells are classified into 41 abnormal cells and 108 normal cells. In [5], a multilevel segmentation method, which is applicable to abnormal nucleus detection on cervical cells, is used to tackle the problems of the segmentation of abnormal nucleus areas and the separation of adhesion situations and cell clusters. Experimental results of [5] show that this method can deliver a high detection accuracy.

**Figure 1:**
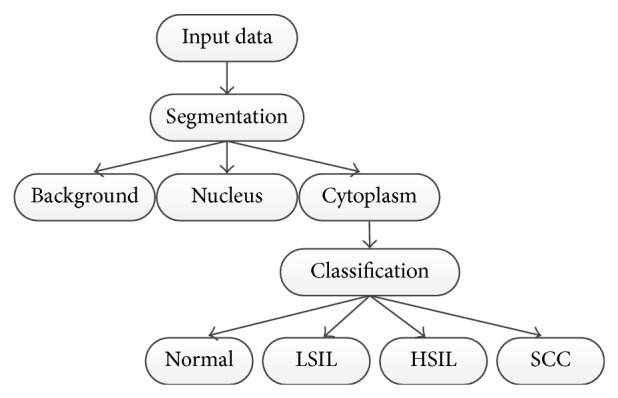
Cell categories. Roughly four categories: normal, low-grade lesion, high-grade lesion, and cancer [9].

In [6, 7], a cervical cancer detection method based on pixel-level top-down feature extraction strategy and svm (Support Vector Machine) feature classification is proposed. In [8], the authors extracted the cell-level morphological and luminosity features for classification, but the segmentation result is not satisfying and may undermine the accuracy of features. In [9], the authors proposed an automatic method for cervical cancer cell segmentation and classification. The authors used their proposed method to classify cervical cells into four classes, that is, normal cells, LSIL (low-grade squamous intraepithelial lesion), HSIL (high-grade squamous intraepithelial lesion), and SCC (squamous cell carcinoma), which are shown in Figure 1.

However, most previous works only took single or a few cell images for analysis and the extracted features and analysis results are restricted to specific application.

In this paper, the images are provided by pathologists, which are used for lesion screening. In pathology domain, cervical cancer can be divided into two categories, that is, cervical adenocarcinoma and cervical squamous cell carcinoma. Compared to cervical adenocarcinoma, cervical squamous cell carcinoma is more common. Clinically, cervical cancer mostly refers to cervical squamous cell carcinoma. This paper is mainly concerned about the research on cervical epithelial cells and 48 pathological images that are taken to the process and analysis in our study.

**Figure 2:**
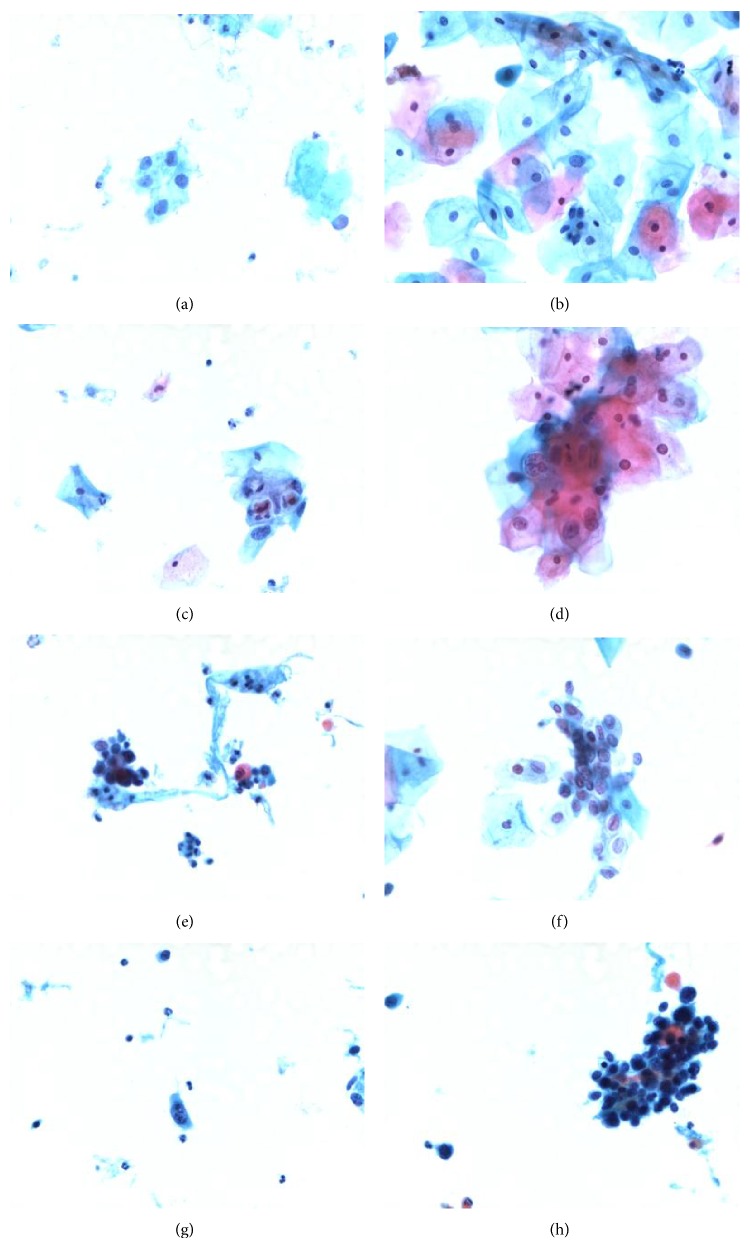
Representation of cervical squamous epithelial cells in different categories of TBS. (a) Normal. (b) ASC-US. (c, d) LISL. (e, f) HISL. (g, h) SCC.

In pathological diagnosis, liquid thin-layer cytology production technology is applied to get cervical smears, from which people can observe conveniently and obtain high-quality microscopic images [10]. In Figure 2, there are many images in different stages. The categories are defined in the Bethesda system (TBS) [11].

**Figure 3:**
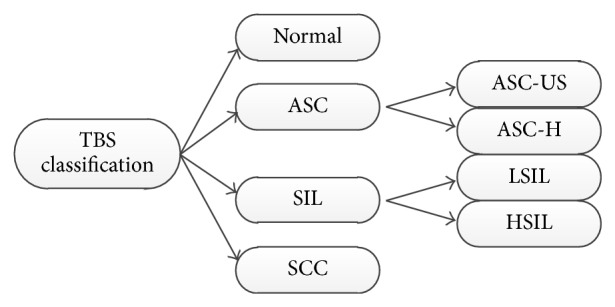
TBS grading of cervical squamous epithelial cells [11].

In this paper, both feature quantification and abnormal detection are based on TBS grading standards and expert diagnosis experiences. According to the lesion degree, TBS classifies cervical squamous epithelial cells into different categories, as shown in Figure 3. Details of TBS grading standards are described below.

1. Normal: normal stage, no lesions.
2. ASC (atypical squamous cell): the subcategories are ASC-US (ASC-undetermined significance) and ASC-H (ASC cannot exclude HSIL).
3. LSIL (low-grade SIL): expanded nucleus, the nucleus is at least three times as big as normal nucleus, with enlarged N/C (ratio between nucleus and cytoplasm), commonly having binucleated and multinucleated conditions, hyperchromatic and in homogeneous distribution, nucleus hyperkeratosis, and cytoplasm jacinth-dyed.
4. HSIL (high-grade SIL): expanded nucleus the same as LSIL, with reduced cytoplasm, more enlarged N/C than LSIL, hyperchromatic, fine or coarse granules are in homogeneous distribution, irregular nucleus boundary, and the existence of nuclear grooves.
5. SCC (squamous cell carcinoma): hyperchromatic and in heterogeneous distribution, as well as meganucleus.

Taking full advantage of images of practical lesion screening and specialists' diagnostic experience can make computer assisted image analysis more valuable.

This paper is conducted under the assistance and instructions of pathologists. They also provide the cervical squamous epithelial cell images. The two main contributions of our study are summarized below.

One is cell feature quantification. Besides the commonly used features, like size, N/C, circularity, compactness, and color strength [12], some features related to pathology need to be extracted, including abnormal morphology, hyperkeratosis, and deeply stained degree. The extracted feature descriptors are related to cervical cell pathological descriptors, making feature parameters more valuable for further analysis.

The other is abnormal cell detection method based on feature quantification. Radiation propagation clustering method [13, 14] is applied to classify abnormal cells into different categories. Moreover, the research on the features of abnormal cells can produce more information.

## 2. Methods

### 2.1. Acquisition of the Image Set

**Figure 4:**
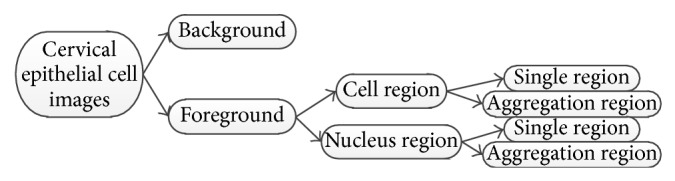
Definition of regions in cell images.

Due to the complexity of pathological cell images, there are many overlapping and aggregated situations, as well as the weak boundary problem caused by uneven dyeing [15–18]. These serious situations may lead to unsatisfactory segmentation outcome. In our study, we apply manual segmentation approach to get the informative regions. The standard of manual segmentation is shown in Figure 4.

**Figure 5:**
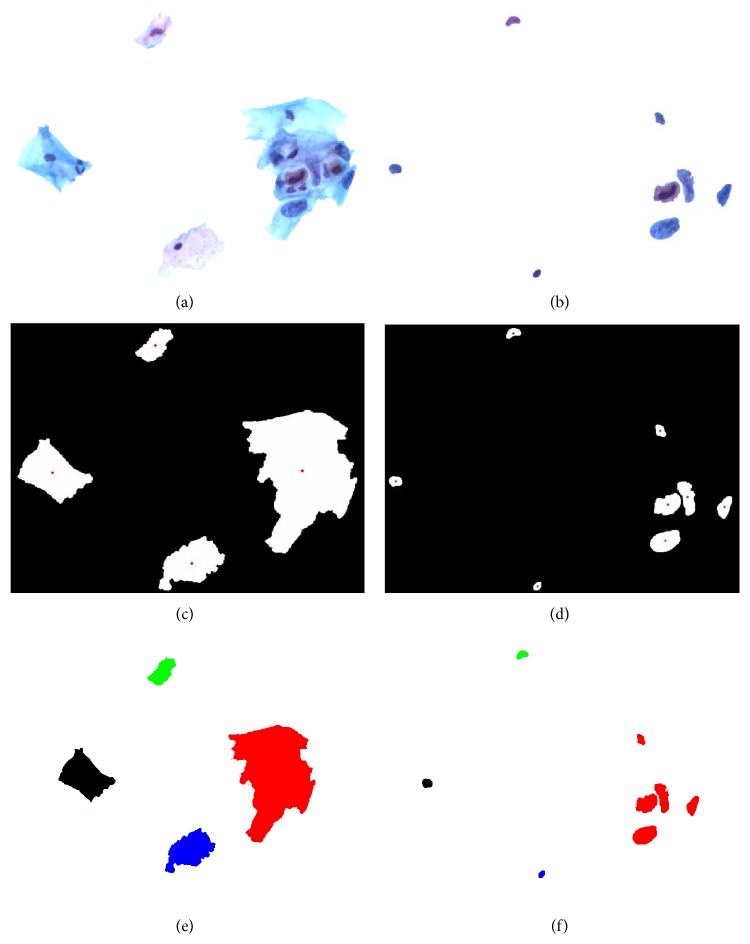
Representation of regions. (a) Cell regions. (b) Nucleus regions. (c) Binary map of cell regions with red points as the cell centroids. (d) Binary map of nucleus regions with red points as the nucleus centroids. (e, f) Region color labeling and the regions in (f) have the same color as (e) have owner-member relationship.

Taking Figure 2(c) image as an example, its related manual segmentation results are shown in Figure 5. The informative sections of Figure 2(c) are the combination of Figure 5(a), the cell regions, and Figure 5(b), the nucleus regions. After morphological dilation, erosion, open, close, and filling operations, the corresponding binary maps [19] can be produced with individual regions as shown in Figures 5(c) and 5(d). The centroids are marked in red and each region is labeled with numbers. Single cell image set and aggregation cell image set are the storage of all the information.

Centroid locations can be used to determine which nucleus region belongs to which cell region. The judgment rule is the minimum distance between the centroid pair. The regions in the same class have the same color label, as shown in Figures 5(e) and 5(f). Cell image sets and nucleus image sets are the foundations of feature extraction.

### 2.2. Quantification of Morphological Difference

The level of difference is calculated mainly by the comparison between abnormal cells and normal cells. In this paper, the level of morphological difference is described by the feature combination of the size of nucleus *A*, N/C *P*, circularity *C*, compactness *E*, centroid position (*x*_0_, *y*_0_), and the nucleus boundary *f*(*x*, *y*). The level of morphological difference can help pathologists detect morphological abnormalities of a single cell and help pathologists determine the lesion areas.

The morphological difference degree can be composed of two parts, which are the size difference degree and the shape difference degree. The size difference degree is described by the size of nucleus and N/C, which is mainly compared to normal cells. The shape difference degree can be described by circularity, compactness, and string distribution shape descriptor.

#### 2.2.1. Size Difference Degree

The ratio between the size of abnormal nucleus and the size of normal nucleus; the ratio between the N/C of abnormal cells and that of normal ones is indicated by ∇*P*. The corresponding equations are written as follows:

(1) $$\begin{array}{c} \nabla A=\frac{A}{A\_\text{normal}},\\ \nabla P=\frac{P}{P\_\text{normal}}, \end{array}$$

where *A*_normal and *P*_normal represent the size and N/C of normal cells, respectively. *A* and *P* represent the size and N/C of detected cells, respectively.

Based on pathology, we have the following.

*Criterion 1.* When the nucleus of the detected cell satisfies the conditions that ∇*P* > *P*_0_ or ∇*A* > *A*_0_, the detected cell can be treated as abnormal and pathologists should not rule out the possibility of lesion for further analysis. ∇*P*_0_ and ∇*A*_0_ represent the thresholds. In this paper, ∇*P*_0_ and ∇*A*_0_ are set to 2 and 2.5, respectively.

#### 2.2.2. Shape Difference Degree

Shape difference degree is used for describing heteromorphic features of nucleus. Nucleuses of normal cells are in regularly circle shape, boat shape, or shuttle shape. Abnormal shapes are observed in lesion situations. Shape difference degree can be depicted in two ways, both of which are related to pathology and described by the following two criteria. 

**Figure 6:**
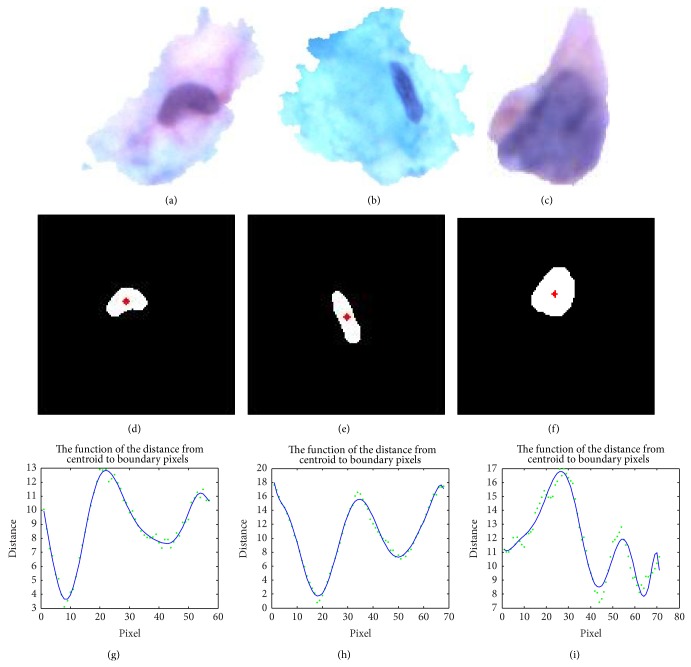
The shape difference descriptor on nucleus. (a, b, c) all are individual cells stored in cell image set. (d, e, f) Corresponding binary maps with red centroid marks. (g, h, i) Point sets and curve fitting.

*Criterion 2.* When the shape of the detected cell satisfies that *C* < *C*_0_ or *E* < *E*_0_, the cell can be determined as an abnormal cell and pathologists cannot rule out the possibility of its being lesion for further analysis. Here, *C*_0_ indicates the circularity of normal nucleus, while *E*_0_ indicates the compactness of normal nucleus. Each value is determined by each weighted average value of a set of normal nucleus and cells. The bigger the set, the higher the reliability of thresholds is. In this paper, *C*_0_ and *E*_0_ are set to 0.8 and 0.7. In Figure 6(a), *C* is 0.7485 and *E* is 0.6667, which satisfy Criterion 2. Therefore, the cell in Figure 6(a) can be judged as the abnormal one. 

*Criterion 3*. When the string distribution of shape descriptor of the detected cell satisfies *N* > *N*_0_, the cell is determined as abnormal.

In this paper, *N*_0_ is set to 4. The string distribution of shape descriptor is based on the descriptor of nucleus boundary. The shape descriptor can be extracted by the following procedure. Given the binary maps, locating the nucleus position (*x*_0_, *y*_0_), to get the nucleus boundary by edge detection algorithm. Starting from a random point in the boundary, the distance *d* between the point (*x*, *y*) and nucleus centroid can be calculated by traversing all points in the boundary.

The distance *d* can be calculated as

(2) $$\begin{array}{c} d=\sqrt{{\left(x_{0}-x\right)}^{2}+{\left(y_{0}-y\right)}^{2}}. \end{array}$$

The distance values can be represented in the Cartesian coordinate. After using a high order polynomial function to fit the points in each plane, the total number *N* of all peaks and valleys in each curve is counted as the string distribution shape descriptor. The bigger *N* is, the more complex the nucleus shape is. From Figure 6(a) to Figure 6(c), *N* values of nucleus are 4, 4, and 6, respectively. In Figure 6(c), the detected cell satisfies Criterion 2, so it can be determined as abnormal.

### 2.3. Hyperkeratosis and Deeply Stained Feature Quantification

The phenomenon that cervical squamous epithelial cells turn to jacinth after dyeing is called hyperkeratosis. It is commonly seen in LSIL condition. From Figure 2(b) to Figure 2(d), we can find that there are many cells in this situation.

Deeply stained nucleus feature is important for lesion identification, especially for the judgment of cells on SCC stage. The color strength *I*(*R*, *G*, *B*) is used for the descriptor of the feature. The strength is defined by the average of *R* (read), *G* (green), and *B* (blue) values. The relationship between the descriptors and pathological judgment can be defined as follows. 

*Criterion 4*. When the color strength *I*(*R*, *G*, *B*) descriptor of the detected cell satisfies {*I*(*R*, *G*, *B*)∣(*R*_*l*0_ < *R* < *R*_*l*0_′)&(*G*_*l*0_ < *G* < *G*_*l*0_′)&(*B*_*l*0_ < *B* < *B*_*l*0_′)}, the cell is determined as abnormal and its TBS grading level may possibly be LSIL. 

*Criterion 5*. When the color strength *I*(*R*, *G*, *B*) descriptor of the detected cell satisfies {*I*(*R*, *G*, *B*)∣(*R* < *R*_*h*0_)&(*G* < *G*_*h*0_)&(*B* < *B*_*h*0_)}, the cell must have a deeply stained phenomenon in most cases and its TBS grading level may possibly be SCC.

In this paper, we set *R*_*l*0_ = 120, *R*_*l*0_′ = 170, *G*_*l*0_ = 70, *G*_*l*0_′ = 140, *B*_*l*0_ = 120, *B*_*l*0_′ = 190, *R*_*h*0_ = 90, *G*_*h*0_ = 90, and *B*_*h*0_ = 190.

### 2.4. Abnormal Detection and Grading on Individual Cells

Based on the features discussed above, a fast abnormal detection method on cervical squamous epithelial cells is proposed. The detection procedure follows the way that when the feature of detected cell satisfies any criterion, the cell is determined as abnormal and pathologists should not rule out the possibility of its being lesion for further analysis.

The cervical squamous epithelial abnormal cells have the traits, including the enlarged nucleus area, enlarged N/C, heteromorphism, deeply stained and hyperkeratosis. Based on the experiences of pathologists, using nucleus area and N/C, most abnormal cells can be easily identified.

In this paper, Criterion 1 is first applied and then the cell is judged by Criteria 4, 5, 2, and 3, successively. Affinity propagation (AP) algorithm is implemented for further analysis on the detected abnormal cells. Aiming at simplifying dataset and performing classification to realize preliminary grading of abnormal cells, the AP algorithm can classify large amount of data directly without the predefined number of classes and preset centers. It is aimed at simplifying dataset and doing further classification based on clustering centers to realize preliminary grading of abnormal cells.

The generated clustering centers can be used as sample centers for further data analysis. Pathologists only need to make further analysis on the sample centers and therefore the screening efficiency can be highly improved. Because different parameters have different weights for identification, these parameters cannot be mixed up.

In our study, we take different features respectively to form sample distance with AP algorithm. Each sample center and corresponding feature threshold can be obtained. Our proposed fast abnormal detection method realizes preliminary grading on abnormal cell samples among three categories, which are LSIL, HSIL, and SCC.

## 3. Experiments and Results

**Figure 7:**
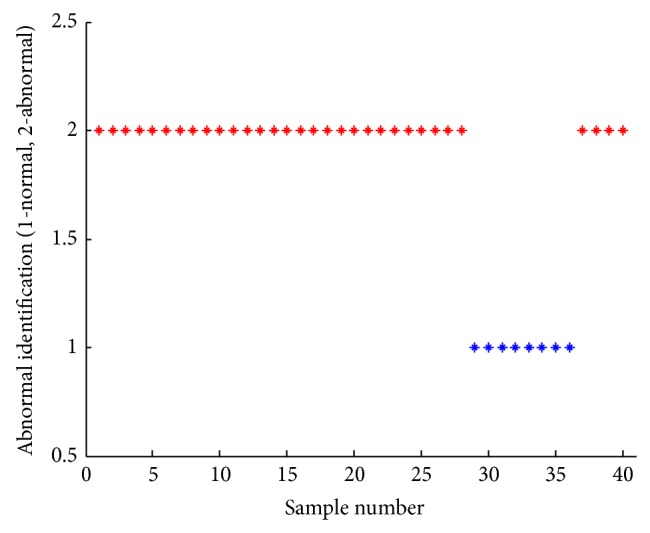
The detection results of individual cells.

From the image sets of cervical squamous epithelial cells, we randomly select 40 cells. After feature quantification on cell images and nucleus images, our proposed fast abnormal detection method is applied to cells classification. The 40 cells are classified into 34 abnormal cells and 4 normal cells. The detection result is shown in Figure 7 and the detection accuracy is 100%. Experimental results show that the abnormal detection method on cervical squamous epithelial cells is efficient and effective.

Applying AP algorithm to the quantified features of the 36 abnormal cells, we get sample centers, which are shown in Tables 1 and 2. The classification thresholds can be set by the results of AP algorithm. In Criterion 1, ∇*P*_0_ = 2 and ∇*A*_0_ = 2.5. In Criterion 2, *C*_0_ = 0.8 and *E*_0_ = 0.7. In Criterion 4, *R*_*l*0_ = 120, *R*_10_′ = 170, *G*_*l*0_ = 70, *G*_*l*0_′ = 140, *B*_*l*0_ = 120, and *B*_*l*0_′ = 190. In Criterion 5, *R*_*h*0_ = 90, *G*_*h*0_ = 90, and *B*_*h*0_ = 190.

The cervical squamous epithelial cells are classified into three categories and the classification results are shown in Table 3. In the detection and identification of abnormal cells, cells in SCC stage can be easily detected, which satisfy the features of deeply stained, enlarged area, and N/C. More specifically, when the parameters of cell features satisfy that *R* < 120, *G* < 120, *B* < 200, ∇*A* > 2, and ∇*P* > 10 at the same time, the detected cell is the abnormal one and is possibly cancer. There are two main differences between LSIL and HSIL. The first is that the N/C of LSIL cells is smaller than that of HSIL cells, while the second is that HSIL cells have heteromorphic features and HSIL cells have deeply stained nucleus phenomenon. Thus, when the parameters of cell features satisfy that *C* < 0.8 and *E* < 0.7 at the same time or ∇*P* < 5, *R* < 170, *G* < 120, and *B* < 200 at the same time, the detected cell is abnormal and its possibility of being in the LSIL stage cannot be ruled out. After the determination of all LSIL and HSIL cells, the rest of undetermined abnormal cells cannot rule out the possibility of being in the HSIL stage.

Based on the experimental results in Table 3, the grading accuracy of abnormal cells is 76.47%. A small amount of misclassification can be tolerable, which is mainly due to two practical facts. First, cells in more severe stages cannot be ruled out the possibility of being in the comparatively less severe stages. In practical application, cells in SCC stage may be classified as HSIL or LSIL cells and cells in HSIL stage may be classified as LSIL cells. Thus, sample 5 and sample 26 are misclassified. Second, cells in less serious stages misclassified into more serious stages make no negative influence on early treatments.

## 4. Conclusion

This paper presents the study on feature quantification and abnormal detection on cervical squamous epithelial cells. Two main aspects are accomplished. First, on the foundation of stored cell image sets and integrating with various feature descriptors, we extract quantified features of individual cells and aggregation cells. These feature descriptors can convert images into data information and are used for building criteria. Second, a fast abnormal cell detection method is proposed. The method takes advantage of clinical experiences and can realize the detection and identification of individual cells. Integrated with pathological experiences about feature quantification and criteria, the detection accuracy is high, but the segmentation and classification of cell images need more substantial work to improve their design and effectiveness.

## Figures and Tables

**(a):** 

Clustering	Distance of samples					
	Criterion: the size of nucleus *A*					
Categories	I	II	III	IV	V	VI
Number of clustering centers	33	8	31	20	27	23
Samples in the class	5, 6, 19, 22	1, 13	4, 7, 10, 12, 14, 18, 21, 28	2, 9, 11, 15, 16, 17, 26, 32	3, 24, 25, 34	29, 30
Scope of feature values	(1.1, 1.4)	(1.7, 1.9)	(2.2, 2.9)	(3.4, 3.9)	(4.0, 4.5)	(5.1, 6.2)
Feature thresholds	∇*A* < 2		2 < ∇*A* < 5		∇*A* > 5	

**(b):** 

Clustering	Distance of samples					
	Criterion: N/C					
Categories	I	II	III	IV	V	VI
Number of clustering centers	17	5	8	26	12	25
Samples in the class	18, 19, 20, 21, 22, 31, 33	15, 32	4, 6, 27, 30	1, 2, 3, 9, 11, 14, 16	13, 24, 29	7, 10, 23, 28, 34
Scope of feature values	(1.7, 4.7)	(8.3, 9.3)	(11.9, 15.2)	(16.5, 19.9)	(21.6, 25.5)	(26.5, 31.1)
Feature thresholds	*C* > 0.8			*C* < 0.8		

**(c):** 

Clustering	Distance of samples			
	Criterion: circularity			
Categories	I	II	III	IV
Number of clustering centers	5	2	24	19
Samples in the class	1, 3, 4, 6, 8, 9, 11, 12, 13, 15, 16, 18, 20, 21, 22, 26, 28	17, 29, 30, 32	7, 10, 14, 25, 27, 33, 34	23, 31
Scope of feature values	[0.8, 0.9]	[0.8, 0.8]	[0.8, 0.8]	[0.5, 0.7]
Feature thresholds	*C* > 0.8		*C* < 0.8	

**(d):** 

Clustering	Distance of samples			
	Criterion: compactness			
Categories	I	II	III	IV
Number of clustering centers	33	29	23	26
Samples in the class	4, 7, 15, 19, 30, 31, 32	1, 6, 17, 28	8, 11, 14, 18, 20, 27, 34	2, 3, 12, 22, 24
Scope of feature values	[0.5, 0.7]	[0.8, 0.8]	[0.8, 0.9]	[0.9, 0.9]
Feature thresholds	*E* < 0.7		*E* > 0.7	

**(a):** 

Clustering	Distance of samples				
	Criterion: the *R* value in RGB color space				
Categories	I	II	III	IV	V
Number of clustering centers	34	14	19	33	7
Samples in the class	1, 2, 10, 24, 25, 23	9, 13, 17, 28, 29, 31, 32	3, 4, 6, 8, 11, 12, 16, 18, 20, 27, 30	5, 21, 22, 26	15
Scope of feature values	[28,55]	[62,84]	[91,111]	[133,154]	[172,176]
Feature thresholds	*R* < 90		90 < *R* < 120	120 < *R* < 170	*R* > 170

**(b):** 

Clustering	Distance of samples			
	Criterion: the *G* value in RGB color space			
Categories	I	II	III	IV
Number of clustering centers	31	2	33	3
Samples in the class	18, 23, 24, 25, 27, 32	1, 10, 21, 22, 28, 34	4, 5, 9, 13, 14, 20, 29	6, 7, 8, 11, 12, 15, 16, 17, 19, 26, 30
Scope of feature values	[41,66]	[70,83]	[95,120]	[126,203]
Feature thresholds	*G* < 70	70 < *G* < 120		*G* > 120

**(c):** 

Clustering	Distance of samples				
	Criterion: the *B* value in RGB color space				
Categories	I	II	III	IV	V
Number of clustering centers	18	25	14	20	19
Samples in the class	23, 24, 27	2, 10, 21, 22, 28, 31, 32, 34	1, 5	4, 9, 13, 16, 26, 29, 33	3, 6, 7, 8, 11, 12, 15, 17, 30
Scope of feature values	[87,110]	[120,140]	[148,167]	[185,199]	[207,252]
Feature thresholds	*B* < 120	120 < *B* < 190			*B* > 200

**Table 3:** Preliminary classification outcome of abnormal cells.

Lesion grading	Outcome of detection and classification	Actual classification	Actual category: LSIL		Actual category: HSIL		Actual category: SCC	
			Misclassified category: HSIL	Misclassified category: SCC	Misclassified category: LSIL	Misclassified category: SCC	Misclassified category: LSIL	Misclassified category: HSIL
LSIL	5, 17, 18, 19, 20, 21, 22, 31, 32, 33	17–22 30–33	—	—	5	—	—	—
HSIL	1, 3, 6, 7, 8, 11, 12, 13, 15, 16, 26, 30	1–16	30	—	—	—	—	—
SCC	2, 4, 9, 10, 14, 23, 24, 25, 27, 28, 29, 34	23–29 34	—	—	—	2, 4, 9, 10, 14	—	26
